# Recovery of influenza A viruses from lake water and sediments by experimental inoculation

**DOI:** 10.1371/journal.pone.0216880

**Published:** 2019-05-15

**Authors:** Daniela Numberger, Carola Dreier, Colin Vullioud, Gülsah Gabriel, Alex D. Greenwood, Hans-Peter Grossart

**Affiliations:** 1 Leibniz Institute for Zoo and Wildlife Research, Berlin, Germany; 2 Heinrich Pette Institute, Leibniz Institute for Experimental Virology, Hamburg, Germany; 3 University of Veterinary Medicine Hannover, Foundation, Hannover, Germany; 4 Leibniz Institute of Freshwater Ecology and Inland Fisheries, Stechlin, Germany; 5 University of Potsdam, Institute of Biochemistry and Biology, Potsdam, Germany; 6 Freie Universität Berlin, Department of Veterinary Medicine, Institute for Virology, Berlin, Germany; Linnaeus University, SWEDEN

## Abstract

Influenza A viruses (IAV) are zoonotic pathogens relevant to human, domestic animal and wildlife health. Many avian IAVs are transmitted among waterfowl via a faecal-oral-route. Therefore, environmental water where waterfowl congregate may play an important role in the ecology and epidemiology of avian IAV. Water and sediment may sustain and transmit virus among individuals or species. It is unclear at what concentrations waterborne viruses are infectious or remain detectable. To address this, we performed lake water and sediment dilution experiments with varying concentrations or infectious doses of four IAV strains from seal, turkey, duck and gull. To test for infectivity of the IAV strains in a concentration dependent manner, we applied cultivation to specific pathogen free (SPF) embryonated chicken eggs and Madin-Darby Canine Kidney (MDCK) cells. IAV recovery was more effective from embryonated chicken eggs than MDCK cells for freshwater lake dilutions, whereas, MDCK cells were more effective for viral recovery from sediment samples. Low infectious dose (1 PFU/200 μL) was sufficient in most cases to detect and recover IAV from lake water dilutions. Sediment required higher initial infectious doses (≥ 100 PFU/200 μL).

## Introduction

Influenza A viruses (IAV) are widespread single stranded negative-sense RNA viruses with a broad host range including birds [1–3], humans [4–7], horses [8–10], pigs [11–13] and marine mammals [14,15]. Waterfowl are the natural reservoirs of IAVs [2,16,17] and avian IAV can cause fatal outbreaks among wild birds and poultry [16–22]. Human infections with avian IAVs demonstrate their zoonotic potential [20,23–31].

Avian IAVs are shed into water by birds in high concentration via faeces [3,32,33]. Once shed, IAVs remain both environmentally persistent and infectious, particularly in cold freshwater (4°C, 17°C) with 0 ppt salinity [3,34,35]. The role of the environment in IAV transmission, particularly water sources used regularly by waterfowl and other bird species is not fully understood. However, there is increasing evidence that water plays an important role in the ecology, epidemiology and transmission of avian IAV [3,24,32,36,37]. Whereas human and other mammalian IAVs are mainly transmitted through smear infection and inhalation of aerosols and droplets [4,38–40], avian IAVs are transmitted via a faecal-oral-route. Water may play an important role in indirect transmission via faecal contamination [3,33,37,41].

Leung et al. [42] indicated that water, such as from drinking troughs, can be used for avian influenza surveillance. Water can be both simultaneously contaminated by multiple strains and infect multiple individuals. This is also true for natural water bodies which are often frequented by waterfowl and consequently contaminated by IAVs. This has been shown for a lake used by an Alaskan dabbling duck population [32] and water bodies along the Atlantic Flyway [43]. Thus, it could be quite useful to include lake water and sediment when conducting IAV surveillance of wild waterfowl populations or drinking water for domestic poultry.

However, the detection of infectious IAVs in water is challenging. There are no standardized methods for the detection and isolation of IAVs from water and sediment samples. In addition to the expected high dilution of virus in water bodies, detection from sediments is complicated by high concentrations of microbes and substances that can interfere with viral culturing experiments, e.g. bacteria and fungi. Sediment might concentrate the virus and viral particles may be protected (e.g. from UV-light) resulting in longer persistence [44–46]. This has been demonstrated in an experiment in which lake sediment, duck faeces and duck meat were inoculated with IAV and persistence was found to be longest in lake sediment [46].

Sixty seven sediment samples from five lakes were screened by influenza virus specific PCR with 26 (= 38.8%) found to be positive (S1 Table). Viral isolation attempted from the PCR positive samples using embryonated chicken eggs and Madin-Darby Canine Kidney (MDCK) cell cultures was, however, unsuccessful. To determine if the lack of cultivation success was due to low viral concentration, dilution experiments were performed with water and sediment using four distinct serially diluted IAV strains. Embryonated chicken eggs and MDCK cell cultures were then performed on the diluted strains to determine the minimal viral concentration/infectious dose needed for IAV detection by cultivation. In addition, we aimed to test the hypothesis that the probability of recovering IAV from inoculated freshwater and sediment samples depends on different predictors including initial sample type, virus concentration, used cultivation method and virus strain by statistical analyses. The results are discussed in the context of the persistence of IAVs in the environment and the applicability of water and sediment samples to IAV surveillance.

## Materials and methods

**Fig 1:**
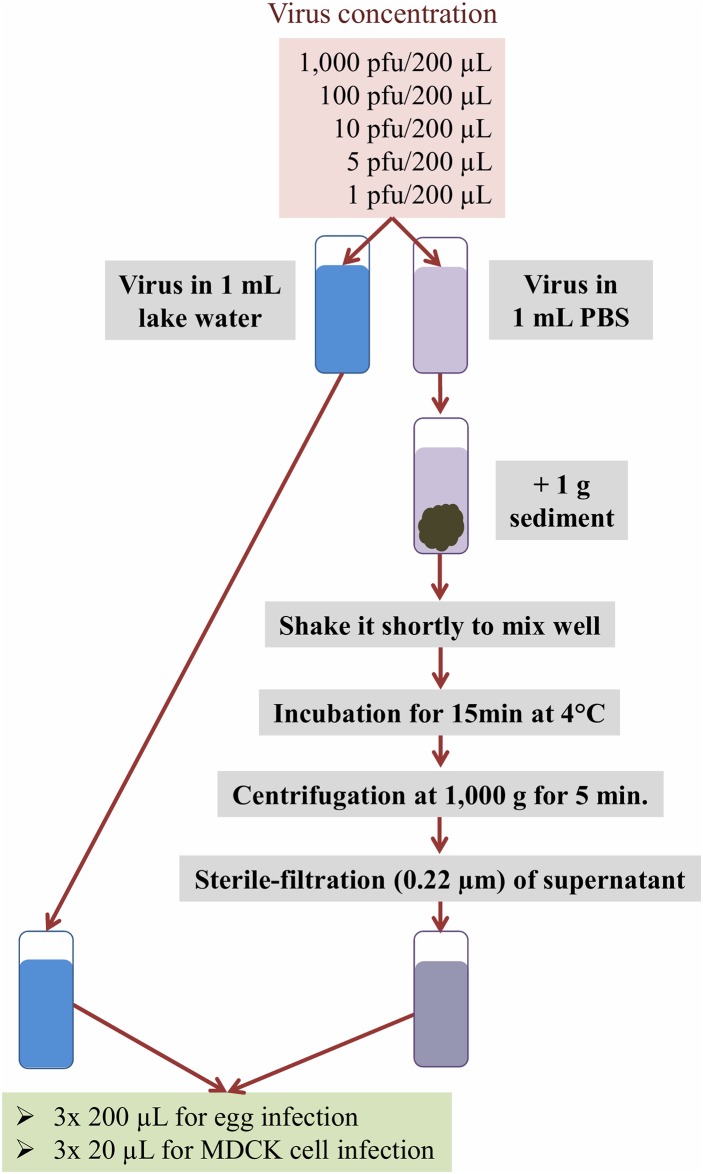
Schematic representation of the dilution experiment design. Lake surface water and sediment were collected on October 16, 2015 from the lake Stechlin (Brandenburg, Germany) and influenza A virus dilutions were made with the collected water. Detection and recovery from inoculated water and sediment samples were performed by cultivation in embryonated chicken eggs and Madin-Darby Canine Kidney (MDCK) cells.

The experimental design is shown schematically in Fig 1. The experiment was designed according to the protocols of virus isolation from beach sand and sediment by Poulson et al. [47] and Dalton et al. [43], respectively. We additionally compared our experimental design to a similar study in which they established a protocol to concentrate and recover influenza A viruses from large volumes of water [48]. Instead of using a non-pathogenic reverse-genetic virus we decided to use four different pathogenic influenza virus strains. We used five dilutions and applied embryonated chicken eggs and MDCK cell culturing as they did to obtain sufficient data.

### Ethics statement

According to the German animal protection law experiments with (11-days) embryonated chicken eggs do not need any specific permission. No animal experiments were performed.

### Sampling of lake water and sediment

Water and sediment samples used for the inoculation experiments were taken from the oligo-mesotrophic Lake Stechlin in Brandenburg, Germany (53°08'56.7", N 13°02'33.5"E) on October 16, 2015. Surface water was collected with sterile 50 mL tubes and the first centimetre of the sandy sediment was obtained using a plexiglass tube (length 50 cm, Ø 44 mm) as a sediment corer. Samples were stored at -20° C. Both sample types were tested negative for IAV by conventional PCR of a 104 bp size fragment of the Matrix gene according to Ward et al. [49]. After reamplification and a total of 70 amplification cycles (S1 Table) all samples were negative. The lake water was sterile-filtered through 0.22 μm Sterivex filters (Merck Millipore, Germany) before use. The moist sediment sample was mixed with a sterile spoon to make it homogeneous and split into 1 g aliquots.

### Influenza A strains and dilutions

The experiment was set up in such a way that IAV negative natural water and sediment representing realistic conditions in which IAVs are transmitted, e.g. an environment in which wild and domestic bird influenza outbreaks occur, were used. Experiments were carried out under S3 laboratory conditions at the Heinrich Pette Institute, Hamburg, Germany. The following IAV strains were tested: A/Seal/Massachusetts/1/1980 (H7N7) [50], A/Turkey/England/1977 (H7N7) [51], A/Gull/Maryland/704/1977 (H13N6) [52] and A/Duck/Alberta/35/1976 (H1N1) [53]. The stock titres of the tested strains were determined via plaque assay on MDCK cells shortly before performing the recovery assay. The viral stocks were diluted in 1 mL sterile filtered lake water to obtain 1000, 100, 10, 5 and 1 plaque forming units (PFU) per 200 μL (volume used for inoculation). Virus dilutions that were used to spike sediment samples were prepared in a final volume of 1 mL as described above but in sterile Dulbeccos’s 1× PBS (Sigma-Aldrich, Merck KGaA, Germany) for each strain and dilution. Then, each 1 mL virus dilution was added to a 1 g sediment aliquot and mixed thoroughly by shaking and inverting the tube. Samples that were not inoculated with influenza A viruses served as negative controls and showed no cytopathogenic effects in MDCK cells nor hemagglutination with 1% chicken erythrocyte suspension. We did not include additional positive controls, since the previously tested virus stocks were all used for artificial infection. To control for strain variation, we used influenza virus strains of different subtypes from different hosts.

### IAV cultivation in embryonated chicken eggs

Specific pathogen free (SPF) eggs were obtained from VALO BioMedia GmbH (Germany) and incubated at 37°C and 55–60% rH for 11 days. Each infection was performed in triplicates (three eggs per sample, for each virus strain and infectious dose) under S3 conditions at the Heinrich Pette Institute, Leibniz Institute for Experimental Virology in Hamburg. 200 μL of each virus dilution was used for infection of eggs. The infected amino-allantoic fluid was then incubated at 37°C for 48 h and analysed subsequently for viral replication by hemagglutination assays.

### Hemagglutination assays

The hemagglutination assay was performed according to Hirst [54] with some modifications. For the assay, 1% chicken erythrocyte suspension was prepared in 0.9% sodium chloride (Th. Geyer GmbH & Co.KG, Germany). Chicken blood was purchased from Lohmann Tierzucht, Cuxhaven, Germany. Spiked samples, negative and positive controls were diluted 2-fold with Dulbeccos’s 1× PBS (Sigma-Aldrich, Merck KGaA, Germany). Fifty μL of 1% erythrocyte solution was added to each virus dilution in a 96-well V-bottom microtiter plate. Hemagglutination was evaluated after incubation for 30 min at 4°C by checking each well for agglutination of red blood cells.

### MDCK cell culture and cytopathogenic effect measurements

A continuous line of Madin Darby canine kidney II (MDCK II) cells was grown in minimal essential medium (MEM, Gibco, Gibco Life Technologies, Germany) supplemented with 10% fetal bovine serum (Invitrogen, Thermo Fisher Scientific, USA), 1% L-Glutamin (Sigma-Aldrich, Merck KGaA, Germany), 1% Penicillin und Streptomycin (Sigma-Aldrich, Merck KGaA, Germany). Cells were infected as described before (modified after Gaush and Smith [55]). Infection of MDCK cells was performed at 37°C for 48 hours in 96-well microtiter plates containing infection medium (MEM with 1% L-Glutamin, 0.2% BSA, 1% each Penicillin, Streptomycin and 1 mg/mL TPCK-trypsin (Sigma-Aldrich, Merck KGaA, Germany)). The viruses were serially diluted to obtain 1, 5, 10, 100 and 1000 PFU. Cytopathic effects were evaluated by light microscopy.

## Results

To ensure that the hemagglutination and cytopathogenic effects observed using experimental IAV dilutions were caused by the introduced IAV laboratory strains and not by viruses in the samples themselves, non-inoculated lake water and sediment samples were included as negative controls. The negative controls did not show any hemagglutination or cytopathogenic effect. They were also all PCR negative. The cultivation results for virus recovery from diluted IAV freshwater and sediment samples are summarized in Table 1.

**Table 1:** Results of spiking experiment to test recovery rate of different IAV strains from water and sediment samples. The cultivation in embryonated chicken eggs was evaluated by the hemagglutination assay. Infection in MDCK cells was shown as cytopathic effect. Green and red colours indicate positive and negative results, respectively.

	WATER												SEDIMENT											
	**Embryonated chicken eggs, haemagglutination assay (HA)**																							
	Seal			Turkey			Duck			Gull			Seal			Turkey			Duck			Gull		
	1	2	3	1	2	3	1	2	3	1	2	3	1	2	3	1	2	3	1	2	3	1	2	3
1,000 PFU																								
100 PFU																								
10 PFU																								
5 PFU																								
1 PFU																								
	**MDCK cells, cytopathic effect (CPE)**																							
	Seal			Turkey			Duck			Gull			Seal			Turkey			Duck			Gull		
	1	2	3	1	2	3	1	2	3	1	2	3	1	2	3	1	2	3	1	2	3	1	2	3
1,000 PFU																								
100 PFU																								
10 PFU																								
5 PFU																								
1 PFU																								

### Sample type

**Fig 2:**
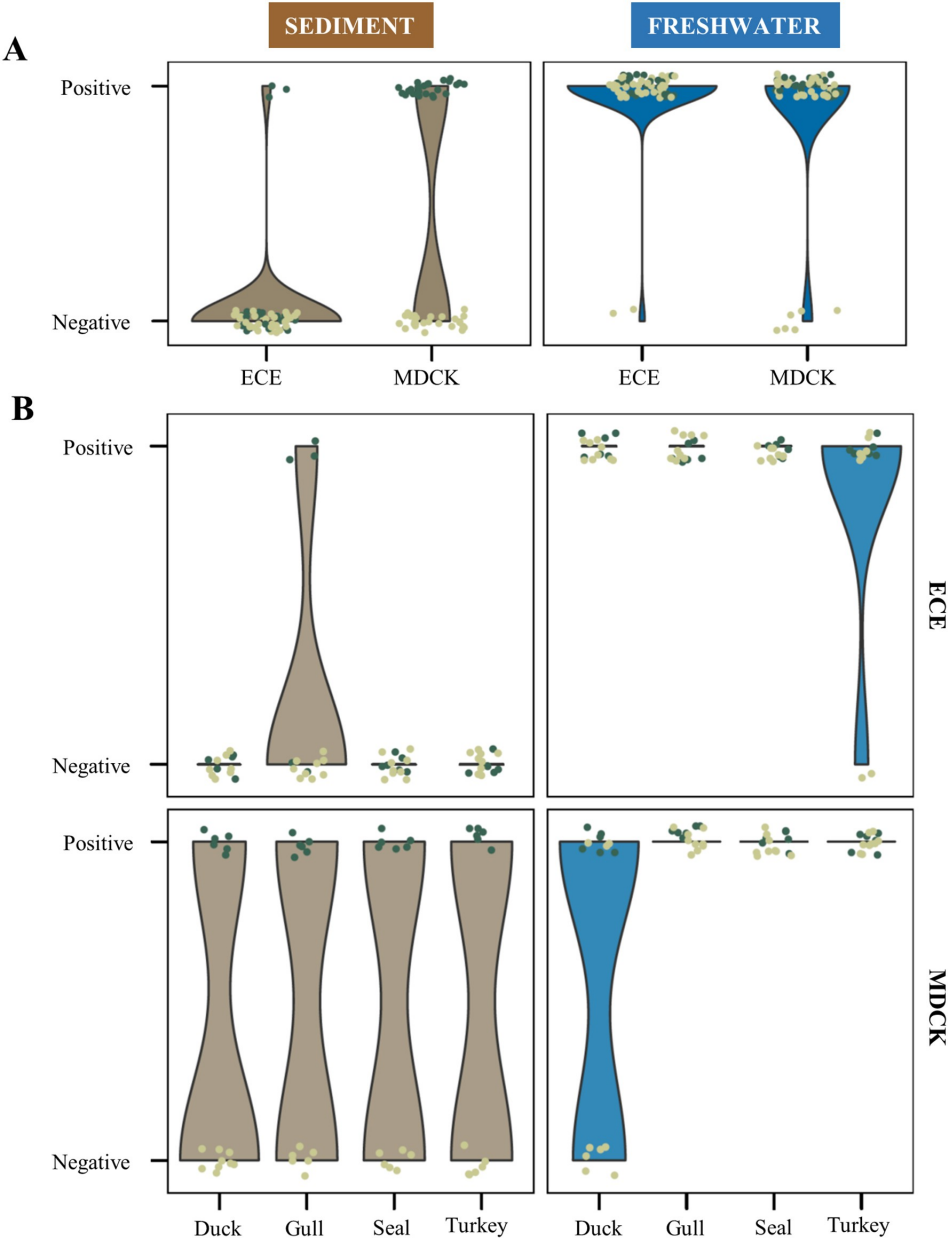
Recovery of influenza A viruses from inoculated sediment and freshwater samples with embryonated chicken eggs (ECE) and Madin-Darby Canine Kidney (MDCK) cells. Panel A graphs show all raw data points including each replicate and their distribution separated by sample type (water compared to sediment) and cultivation method (embryonated chicken eggs compared to MDCK cells). Panel B graphs show the same comparisons but separated by strain. Dark green circles represent samples with infection doses ≥ 100 PFU and light green < 100 PFU. Culture positive or negative data points are indicated on the y-axis. Results of sediment samples are shown in the left panels and of freshwater samples on the right. Influenza A strains are indicated on the x-axis.

**Fig 3:**
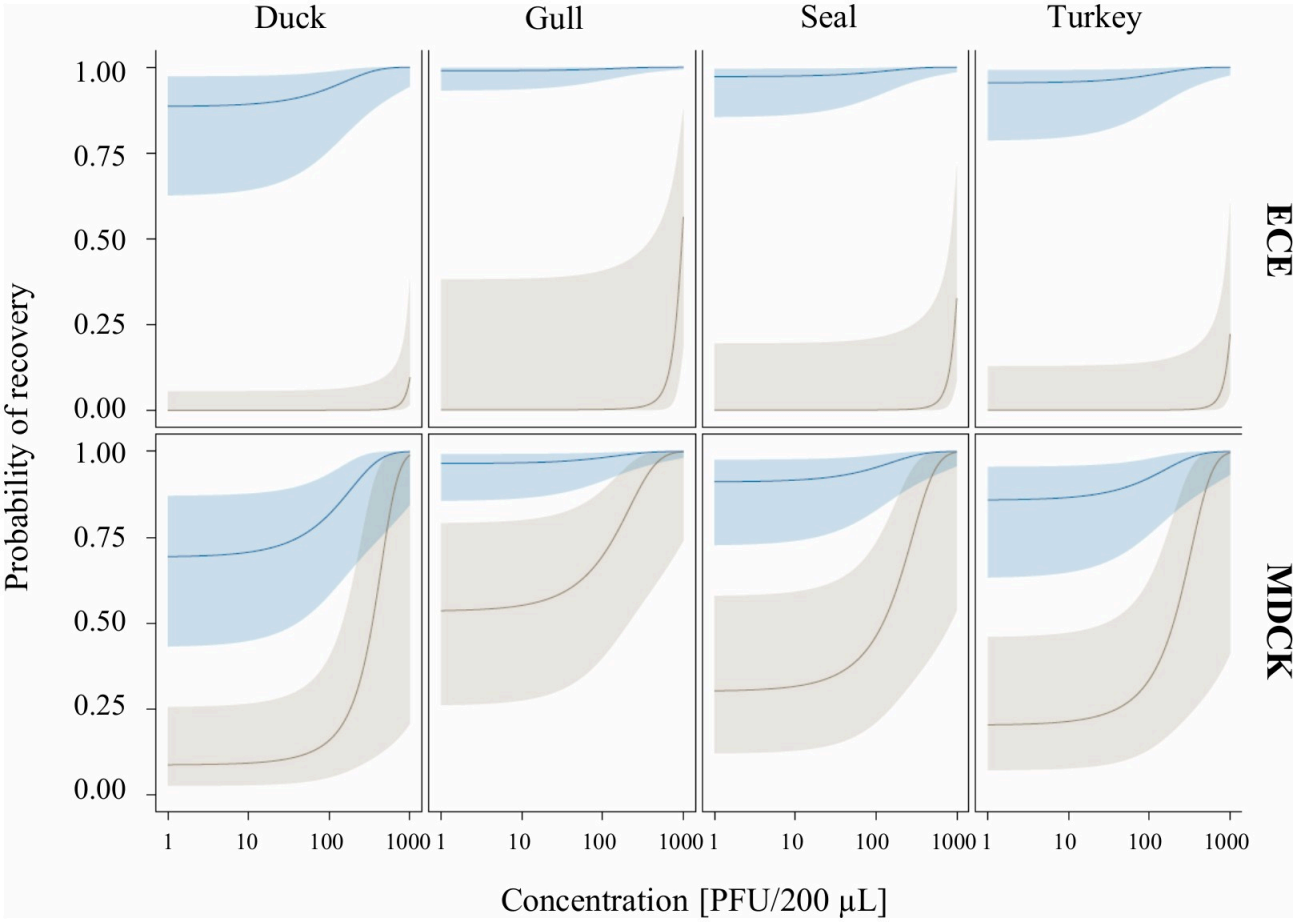
Predicted probability of recovering different influenza A virus strains from inoculated sediment (brown) and freshwater (blue) samples by from embryonated chicken eggs (ECE) and Madin-Darby Canine Kidney (MDCK) cells. Virus concentration (infection dose) is shown on a log scale on the x-axis and the predicted probability is shown on the y-axis. The dark lines indicate the predicted probability of recovery with increasing initial virus concentration. The blue and brown distributions around the lines represent the 95% confidence intervals.

Generally, virus recovery by culture from sediment was significantly less efficient than from freshwater (χ2 = 174.69, p < 0.001, Figs 2A and 3) using both embryonated chicken eggs (log-odd = 11.18) and MDCK cell cultures (log-odd = 3.17).

### Cultivation method

MDCK cells and embryonated chicken eggs have different effects on the probability of recovery in freshwater and in sediment (χ2 = 27.68934, p < 0.001, Figs 2A and 3). In freshwater, embryonated chicken eggs were more efficiently for recovery than MDCK cells (log-odd = 1.23), while for sediment samples the MDCK cells worked better (log-odd = 6.78).

For sediment samples, all infected eggs, except for three eggs infected with the A/Gull/Maryland/704/1977 (H13N6) strain, were negative, whereas recovery was successful in MDCK cell cultures with ≥ 100 PFU/100 μL (Table 1, Fig 2A).

For freshwater samples, both cultivation systems worked well with a higher probability of recovery in embryonated chicken eggs, as the duck strain A/Duck/Alberta/35/1976 (H1N1) was not recovered from infectious doses lower than 10 PFU/200 μL when using MDCK cells (Table 1, Fig 2).

### Influenza virus strains and infectious dose

Strains (χ2 = 12.077, p = 0.007) and the infectious dose (χ2 = 39.512, p > 0.001) also had significant effects on the probability of recovering IAVs (Fig 3). Recovery from sediment samples was only successful when having infectious doses of ≥ 100 PFU/200 μL, whereas in freshwater samples also 1 PFU/200 μL was sufficient to be recovered. In general, the probability of recovery increased with higher infectious doses.

On a descriptive level, a different probability of recovery was observed for the strains A/Gull/Maryland/704/1977 (H13N6) and A/Duck/Alberta/35/76 (H1N1). In freshwater, for example, strain A/Duck/Alberta/35/1976 (H1N1) showed a more efficient recovery in embryonated chicken eggs than in MDCK cells and A/Gull/Maryland/704/1977 (H13N6) was the only strain that had a small chance to be recovered from sediment samples when using embryonated chicken eggs.

## Discussion

Influenza A virus sequences could be detected by PCR from environmental samples, but subsequent attempts to isolate IAVs failed (S1 Table). This result did not determine, whether the PCR detected virus remained infectious, nor indicate which viral strain was detected. Therefore, dilution experiments were undertaken to determine the minimal infectious dose for four different IAV strains necessary to successfully cultivate IAV from water and sediment. We tested statistically, whether the recovery of IAV from inoculated water and sediment samples were dependent on the sample type, infectious dose, strain and cultivation method used.

Significant differences in the culturing efficiency among used IAV strains were observed (Figs 2 and 3). The IAV duck strain A/Duck/Alberta/35/76 (H1N1), for instance, grew better in embryonated chicken eggs than in MDCK cells, which is consistent with a higher receptor-binding affinity to avian cell surface receptors [56–58]. In contrast, the seal strain A/Seal/Mass/1/80 (H7N7) showed little differences in growth in MDCK cells compared to embryonated chicken eggs. However, caution is necessary in the interpretation of these findings given the few strains and experiments performed.

Virus isolation from sediment mostly failed in embryonated chicken eggs and was only possible at high infectious doses in MDCK cells. Although previous studies have shown that virus isolation is more efficient in embryonated chicken eggs than in MDCK cells for both swine and avian IAV [59,60], MDCK cells were more tolerant when using sediment, which may contain many additional contaminants compared to the cleaner water. For example, humic acids and heavy metals may impair the cultivation in embryonated chicken eggs, but may have a smaller effect on MDCK cells. It is known that substances in soil and sediment often inhibit PCR reactions and/or reduce extraction efficiency [61–63]. Bacteria and fungi could also interfere, but are diminished or at least reduced by sterile filtration through 0.22 μm filters. However, a chemical inhibition by any inhibitory compounds, e.g. humic acids cannot be fully excluded. Negative control experiments with IAV free water and sediment did not exhibit viral, bacterial or fungal growth indicating that if any inhibition occurred, it was not of microbial origin.

The low recovery rate from sediment cultures indicates that either there was inhibition, a disruption of virus particles or that most of the virus remained bound to the sediment and were not successfully transferred into the supernatant which was then used for infection. Rapid and tight attachment to sediment or mineral surfaces has been shown previously for different viruses [64–66]. Furthermore, it could be demonstrated that sediment can prolong viral survival [44,45,67]. IAV persistence has been shown to be highest in lake sediment followed by faeces and duck meat [46]. Thus, notwithstanding cultivation difficulties, sediment might be a viral vector and a good sample source for measuring IAV diversity, epidemiology and ecology.

Experimentally IAV diluted water samples demonstrated that minimal infectious doses (1 PFU/200 μL) are needed to infect embryonated eggs or MDCK cells. Previous work has shown that IAV particles are persistent and infectious in freshwater over an extended time, e.g. over 30 days in non-chlorinated river water at 0°C [3] or 100 days at 17°C with salinity of 0 ppt and pH 8.2 [35]. The extended stability and low viral concentration needed to seed infection suggests that lake water and sediments could be involved or could enhance IAV intra and interspecies transmission.

We conclude that IAV cultivation from water samples requires minimal viral titres, but cultivation efficiency is influenced by viral strain and is enhanced in embryonated chicken eggs. Sediment appears to be a source of IAV as well, but further methodological development is needed to improve cultivation efficiency. Our initial PCR screening of sediment samples from different lakes indicated the presence of IAV in 38.8% of samples (S1 Table). However, the inability to culture virus and the findings of the experimental inoculation suggests that the samples had either viral concentration below the minimum needed to infect embryonated chicken eggs or that the virus particles were degraded due to transport and a freeze-thaw-cycle and were no longer infectious. Although IAV surveillance could benefit from environmental sampling, further methodological development will be required to determine the effect of sample type and length of time between viral shedding and sample collection on the ability to cultivate IAV. The results of the current study suggest that such research is urgently warranted to better understand the spreading and reassortment of new influenza virus strains.

## Supporting information

S1 Table Detection of influenza A in sediment samples by conventional PCR. The first and 3rd-4th centimetre of sediment was obtained using a plexiglas tube (length 50 cm, Ø 44 mm) and ruler as a sediment corer. Samples were stored at -20°C till extraction. RNA from sediment samples were extracted using the ZR Soil/Fecal RNA MicroPrep (ZymoResearch). The RNA was then transcribed in cDNA using the SuperScript^™^ III Reverse Transcriptase (Invitrogen) and 10μL of extracted RNA. The second DNA strand was synthesized with the Klenow DNA Polymerase I (New England Biolabs). Conventional PCR of a 109 bp fragment of the matrix gene running 35 cycles was performed according to Ward et al., 2004 (10.1016/S1386-6532(03)00122-7). Only after a reamplification with another 30 cycles using 1 μL of the PCR product the amplified product was visible on a 1.5% agarose gel stained with Midori Green Direct (Biozym). Isolation of IAV from some of the PCR positive sediment samples using embryonated chicken eggs and MDCK cell cultures as described in the current study failed.
(DOCX)
