# Correction: Recovery of influenza A viruses from lake water and sediments by experimental inoculation

**DOI:** 10.1371/journal.pone.0218882

**Published:** 2019-06-19

**Authors:** Daniela Numberger, Carola Dreier, Colin Vullioud, Gülsah Gabriel, Alex D. Greenwood, Hans-Peter Grossart

There are errors in the author affiliations. The correct affiliations are as follows:

Daniela Numberger^1^, Carola Dreier^2^, Colin Vullioud^1^, Gülsah Gabriel^2,3^, Alex D. Greenwood^1,4^, Hans-Peter Grossart^5,6^

**1** Leibniz Institute for Zoo and Wildlife Research, Berlin, Germany, **2** Heinrich Pette Institute, Leibniz Institute for Experimental Virology, Hamburg, Germany, **3** University of Veterinary Medicine Hannover, Foundation, Hannover, Germany, **4** Freie Universität Berlin, Department of Veterinary Medicine, Institute for Virology, Berlin, Germany, **5** Leibniz Institute of Freshwater Ecology and Inland Fisheries, Stechlin, Germany, **6** University of Potsdam, Institute of Biochemistry and Biology, Potsdam, Germany.
