# Supplementary material for: Recovery of influenza A viruses from lake water and sediments by experimental inoculation
Source: PLoS One. 2019 May 15;14(5):e0216880. doi: 10.1371/journal.pone.0216880 (PMC6519785; doi:10.1371/journal.pone.0216880)
Supplement: S1 Table — The first and 3rd-4th centimetre of sediment was obtained using a plexiglas tube (length 50 cm, Ø 44 mm) and ruler as a sediment corer. Samples were stored at -20°C till extraction. RNA from sediment samples were extracted using the ZR Soil/Fecal RNA MicroPrep (ZymoResearch). The RNA was then transcribed in cDNA using the SuperScript™ III Reverse Transcriptase (Invitrogen) and 10μL of extracted RNA. The second DNA strand was synthesized with the Klenow DNA Polymerase I (New England Biolabs). Conventional PCR of a 109 bp fragment of the matrix gene running 35 cycles was performed according to Ward et al., 2004 (10.1016/S1386-6532(03)00122-7). Only after a reamplification with another 30 cycles using 1 μL of the PCR product the amplified product was visible on a 1.5% agarose gel stained with Midori Green Direct (Biozym). Isolation of IAV from some of the PCR positive sediment samples using embryonated chicken eggs and MDCK cell cultures as described in the current study failed. (DOCX) [file pone.0216880.s001.docx]

**Table S1:** Detection of influenza A in sediment samples by conventional PCR.

| **Lake** | **Stechlin** | | | **Dagowsee** | | | | | **Müggelsee** | | | **Weißer See** | | | **Haussee** | | |
| --- | --- | --- | --- | --- | --- | --- | --- | --- | --- | --- | --- | --- | --- | --- | --- | --- | --- |
| **Site** | **1** | **2** | **3** | | **1** | **2** | **3** | **1** | | **2** | **1** | | **2** | **1** | | **2** | **3** |
| **October 2015** | | | | | | | | | | | | | | | | | |
| **1 cm** | - | **+** | - | | - | - | - | - | | **+** | - | | **‡** | **+** | | **+** | - |
| **3-4 cm** | NA | NA | NA | | - | NA | - | - | | **+** | NA | | **+** | - | | - | **+** |
| **January 2016** | | | | | | | | | | | | | | | | | |
| **1 cm** | - | - | - | | - | - | - | **+** | | - | **‡** | | **‡** | **+** | | **+** | **+** |
| **3-4 cm** | - | - | **+** | | NA | NA | **+** | **+** | | NA | - | | - | - | | **+** | **+** |
| **April 2016** | | | | | | | | | | | | | | | | | |
| **1 cm** | - | - | **+** | | - | - | **+** | - | | **+** | - | | **‡** | - | | - | - |
| **3-4 cm** | - | **+** | - | | - | NA | **+** | - | | - | - | | **+** | - | | NA | NA |

‡ indicates confirmation by sequencing and BLAST search. PCR products were purified and Sanger sequenced on a 3130xl Genetic Analyzer using the BigDye^®^ Terminator v1.1 Cycle Sequencing Kit according to the manufacturer’s instructions (Applied Biosystems, CA, USA).

The first and 3^rd^-4^th^ centimetre of sediment was obtained using a plexiglas tube (length 50 cm, Ø 44 mm) and ruler as a sediment corer. Samples were stored at -20°C till extraction. RNA from sediment samples were extracted using the ZR Soil/Fecal RNA MicroPrep™ (ZymoResearch). The RNA was then transcribed in cDNA using the SuperScript™ III Reverse Transcriptase (Invitrogen) and 10µL of extracted RNA. The second DNA strand was synthesized with the Klenow DNA Polymerase I (New England Biolabs). Conventional PCR of a 109 bp fragment of the matrix gene running 35 cycles was performed according to Ward et al., 2004 (doi: 10.1016/S1386-6532(03)00122-7). Only after a reamplification with another 30 cycles using 1 µL of the PCR product the amplified product was visible on a 1.5% agarose gel stained with Midori Green Direct (Biozym). Isolation of IAV from some of the PCR positive sediment samples using embryonated chicken eggs and MDCK cell cultures as described in the current study failed.
